# Validation of telemedicine-based self-assessment of vital signs for patients with COVID-19: A pilot study

**DOI:** 10.1177/1357633X211011825

**Published:** 2021-05-09

**Authors:** Nobuyuki Kagiyama, Makoto Hiki, Yuya Matsue, Tomotaka Dohi, Wataru Matsuzawa, Hiroyuki Daida, Tohru Minamino, Takatoshi Kasai

**Affiliations:** 1 Department of Digital Health and Telemedicine R&D, Juntendo University, Japan; 2 Department of Cardiovascular Biology and Medicine, Juntendo University, Japan; 3 Department of Emergency Medicine, Juntendo University, Japan; 4 Ogino Memorial Laboratory, Nihon Kohden Corporation, Japan; 5 Cardiovascular Respiratory Sleep Medicine, Juntendo University, Japan

**Keywords:** Telemedicine, COVID-19, information and communication technology, non-contact monitoring, respiratory monitoring

## Abstract

**Introduction:**

In the ongoing COVID-19 pandemic, the development of a system that would prevent the infection of healthcare providers is in urgent demand. We sought to investigate the feasibility and validity of a telemedicine-based system in which healthcare providers remotely check the vital signs measured by patients with COVID-19.

**Methods:**

Patients hospitalized with confirmed or suspected COVID-19 measured and uploaded their vital signs to secure cloud storage. Additionally, the respiratory rates were monitored using a mat-type sensor placed under the bed. We assessed the time until the values became available on the Cloud and the agreements between the patient-measured vital signs and simultaneous healthcare provider measurements.

**Results:**

Between 26 May–23 September 2020, 3835 vital signs were measured and uploaded to the cloud storage by the patients (*n*=16, median 72 years old, 31% women). All patients successfully learned how to use these devices with a 10-minute lecture. The median time until the measurements were available on the cloud system was only 0.35 min, and 95.2% of the vital signs were available within 5 min of the measurement. The agreement between the patients’ and healthcare providers’ measurements was excellent for all parameters. Interclass coefficient correlations were as follows: systolic (0.92, *p*<0.001), diastolic blood pressure (0.86, *p*<0.001), heart rate (0.89, *p*<0.001), peripheral oxygen saturation (0.92, *p*<0.001), body temperature (0.83, *p*<0.001), and respiratory rates (0.90, *p*<0.001).

**Conclusions:**

Telemedicine-based self-assessment of vital signs in patients with COVID-19 was feasible and reliable. The system will be a useful alternative to traditional vital sign measurements by healthcare providers during the COVID-19 pandemic.

## Introduction

Coronavirus disease (COVID-19) is the most important topic in the world. This disease, first identified in late December 2019, had already infected more than 70 m people, and more than 1.5 m people had died of this disease by the end of 2020.^1^ This highly infectious virus spreads through respiratory droplets, contact routes and, possibly, aerosol particles,^2–4^ causing fatal pneumonia not only in elderly and frail patients but also in young and healthy people.^5–7^ Healthcare providers are not an exception; notably, a number of doctors and medical staff have contracted and passed away due to COVID-19.^8^ Another fatal characteristic of the disease is that it leads to sudden respiratory failure, which sometimes precedes the onset of severe symptoms.^9^ Given this nature, close monitoring of the vital signs, such as body temperature and peripheral oxygen saturation, is mandatory for all patients, even those with mild symptoms. Some medical institutes have adopted the methodology of self-assessment of vital signs using telemedicine technologies for patients with relatively mild symptoms in order to reduce the physical contact between the healthcare providers and patients.

Telemedicine refers to the remote delivery of healthcare services using information and communication technologies, thus eliminating the need for physical contact between the healthcare providers and patients.^10^ The most common type of telemedicine device in the care of COVID-19 is a device that uploads the patient-derived medical information so that healthcare providers can remotely check the patient’s health status.^11,12^ However, the majority of these devices are designed for home monitoring by patients in the chronic phase with a good level of activities of daily living; they have not been validated against the standard measurements by trained medical staff in a scientific manner, especially for patients with acute illness. In addition, the feasibility of the devices in hospital settings is yet to be investigated. Thus, the aim of the present study was to elucidate the feasibility and validity of a telemedicine-based system in which patients hospitalised with acute COVID-19 measure and upload their vital signs to a cloud system.

### Methods and materials

We conducted a single-centre observational study that included consecutive patients who were admitted to the Juntendo University Hospital due to suspected or confirmed COVID-19 and who were given telemedicine devices from May–September 2020. Patients with dementia, those admitted to the intensive care unit, and those who were unable to measure their own blood pressure using a manometer were excluded from the study. Using a commercially available digital manometer (UA-651BLE: A&D Medical, Tokyo, Japan), a digital thermometer (C217: TERUMO, Tokyo, Japan), and a pulse oximeter (SP2; TERUMO, Tokyo, Japan), patients measured their vital signs and uploaded the data through the LAVITA gateway (Nihon Kohden, Tokyo, Japan). This system semi-automatically uploads the data to secured cloud storage; the digital manometer automatically transfers the data to the gateway via Bluetooth; and the other parameters, body temperature, and peripheral oxygen saturation (SpO_2_) are manually transferred to the gateway by holding the thermometer and pulse oximeter on the gateway. The uploaded data are referred to only by the authorised healthcare providers and researchers. These measurements were compared with standard measurements performed simultaneously by healthcare providers. The respiratory rates were automatically and continuously measured using a mat-type air pressure sensor placed under the bed mattress (Kaigolog Med: Liquid Design Systems Inc., Tokyo, Japan). The sensor continuously analyses subtle changes in the pressure due to respiratory motions to calculate respiratory rates, and the acquired data are automatically uploaded through an iPad application. While the sensor automatically and continuously reported the respiratory rate, healthcare providers manually measured the respiratory rate by visual assessment several times a day in order to compare with the data acquired by the sensor. All patients attended a 10-minute lecture on the usage of the devices.

The systems uploaded data using a Wi-Fi network provided by the Juntendo University Hospital. The hospital provides an encrypted private Wi-Fi network that patients can use with entering a password for which multiple routers are set on corridors on every floor as well as personal Wi-Fi networks that use routers set inside the hospital rooms.

The primary outcome of the study was the agreement between the data uploaded by the patients and the data measured by the healthcare providers. We also investigated the duration of time taken from when the time vital signs are measured by patients to the time they are available on the Web page where the healthcare providers can check them remotely.

#### Statistical analysis

The data are presented as mean ± standard deviation or median (1st and 3rd quartiles) for continuous variables and as frequency (percentage) for categorical variables. The difference in time to be available online among the parameters was assessed using a one-way analysis of variance. The concordance between the measurements by patients and those by healthcare providers was assessed using intraclass correlation coefficients (ICCs) and Bland-Altman plots. All statistical analyses were performed with R version 4.0.2 (The R Foundation for Statistical Computing, Vienna, Austria) with the packages ‘irr’ and ‘BlandAltmanLeh’.^13,14^ A two-tailed *p*-value of <0.05 indicated statistical significance.

### Results

**Figure 1.**
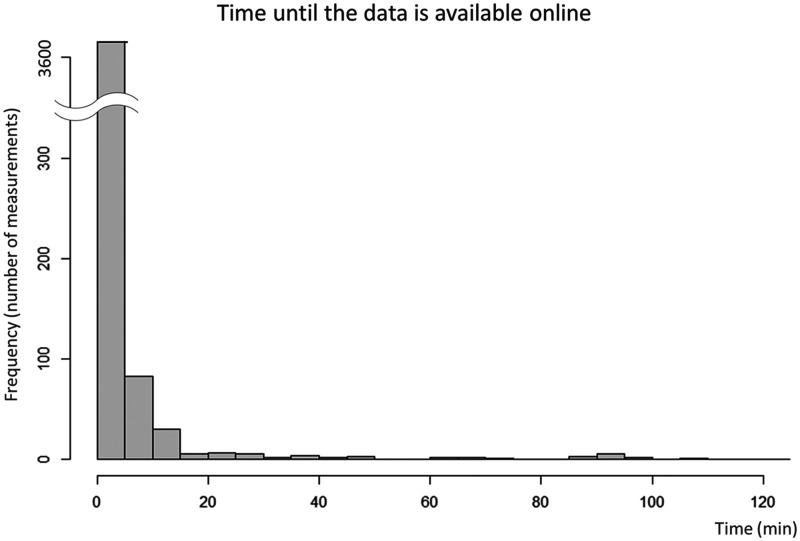
Histogram for the time until the data is online. The *x* and *y* axes show the time until the data become available online and the number of measurements, respectively. The median time until the measurements were available on the cloud system was only 0.35 min. The majority of the data (>95%) were available within 5 min of the measurement.

During the study period, a total of 3835 vital signs (793 SpO_2_, 1582 values of blood pressure and heart rate, and 1460 values of body temperature) were measured and uploaded to the cloud storage by the patients (*n*=16, 71.5 (52.5–74.5) years old, 31% women, 62% with confirmed COVID-19). Background characteristics are summarised in Table 1. All patients had normal sinus rhythm, and 31% and 12% had hypertension and diabetes, respectively. As the respiratory rates were continuously monitored only when the patients were on the bed, the number of respiratory rate measurements was not counted. The median duration from the time when the patients measured the vital signs to the time when the measurements were available on the cloud system was only 0.35 (interquartile range 0.22–0.90) min. This duration was not statistically different among the different vital sign parameters (*p*=0.73). Notably, 95.2% of the measurements were available within 5 min of the patients’ measurements (Figure 1).

**Table 1.** Patient characteristics.

	*n*=16
Age, years	71.5 (52.5–74.5)
Female	5 (31%)
BMI, kg/m^2^	22.6 (19.4–25.8)
COVID-19	
Confirmed	10 (62%)
Suspected	6 (38%)
Heart rhythm	
Sinus rhythm	16 (100%)
Hypertension	5 (31%)
Diabetes mellites	2 (12%)

BMI: body mass index; COVID-19: coronavirus disease.

**Figure 2.**
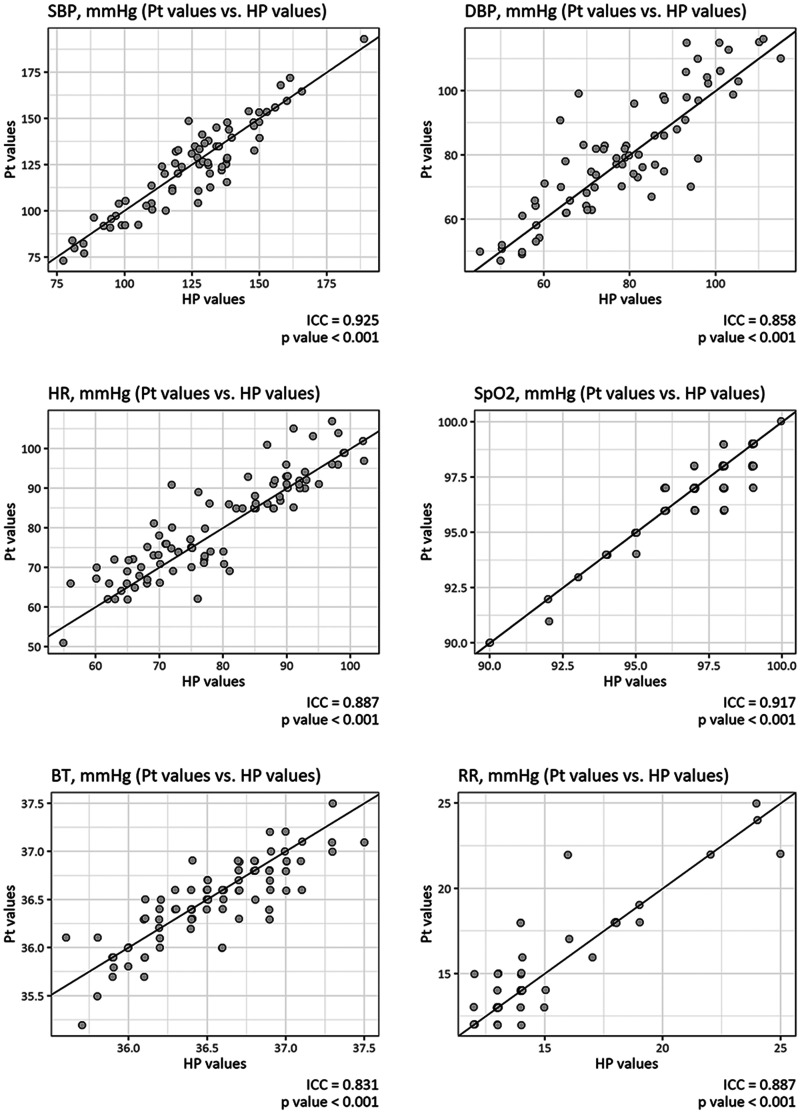
Concordance of measurements by patients and healthcare providers. The self-measured values of vital signs by patients showed excellent concordance with the values measured by healthcare providers. The intraclass coefficient correlations (ICCs) ranged from 0.83–0.93. BT: body temperature; DBP: diastolic blood pressure; HP: healthcare provider; HR: heart rate; Pt: patient; RR: respiratory rate; SBP: systolic blood pressure; SpO_2_: peripheral oxygen saturation.

**Figure 3.**
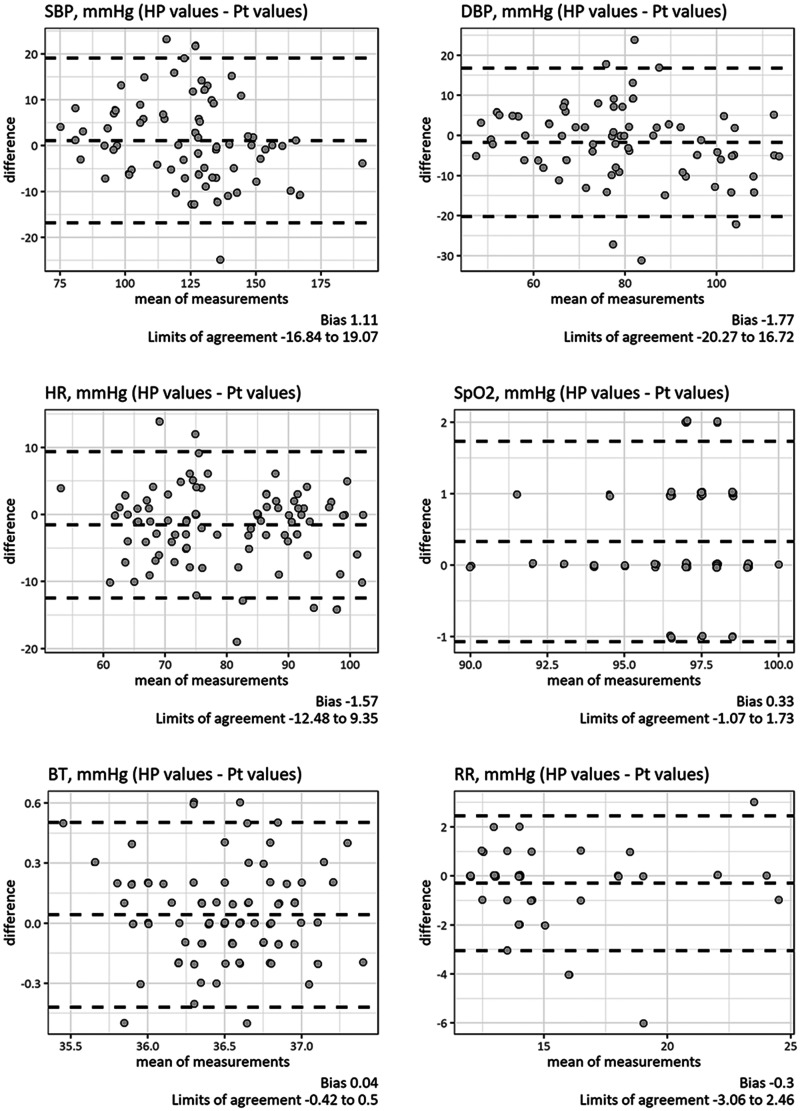
Bland-Altman plots for vital sign measurements. Bland-Altman plots showed no systematic error or bias in the measurements. All the parameters showed clinically acceptable agreement limits. BT: body temperature; DBP: diastolic blood pressure; HP: healthcare provider; HR: heart rate; Pt: patient; RR: respiratory rate; SBP: systolic blood pressure; SpO_2_: peripheral oxygen saturation.

Of these measurements, 483 vital signs (76 systolic blood pressures, 76 diastolic blood pressures, 92 heart rates, 91 SpO_2_s, 88 body temperatures and 60 respiratory rates) were double-checked with simultaneous measurements by healthcare providers. ICC analyses demonstrated that the measurements by the patients and those by healthcare providers, regardless of the parameter, were in excellent agreement. ICCs and their confidence intervals (CIs) were 0.93 (95% CI 0.88–0.95, *p*<0.001) for systolic blood pressures, 0.86 (95% CI 0.78–0.91), *p*<0.001) for diastolic blood pressures, 0.89 (95% CI 0.83–0.92, *p*<0.001) for heart rates, 0.92 (95% CI 0.88–0.94), *p*<0.001) for SpO_2_, 0.83 (95% CI 0.75–0.89, *p*<0.001) for body temperatures and 0.89 [95% CI 0.82–0.93, *p*<0.001] for respiratory rates (Figure 2, panel (a) to (e)). No systematic error was found in the Bland-Altman plots, and all parameters showed clinically acceptable limits of agreement: –17 to +19 mm Hg for systolic blood pressures, –20 to +17 mm Hg for diastolic blood pressures, –12 to +9/min for heart rates, –1 to +2% for SpO_2_, –0.4 to +0.5 degrees for body temperature, and –3 to +2/min for respiratory rates (Figure 3, panels (a) to (e)).

### Discussion

In the present study, we showed that (a) the telemedicine-based self-vital sign check system was easy enough that all including elderly patients (56% were over 70 years old) successfully learned how to use the device from a 10-minute lecture without any trouble, even in the acute phase of illness, (b) the telemedicine-based system was feasible, with most of the measurements available immediately on the cloud system even in an acute care hospital, and (c) self-measurements by patients showed excellent agreements with standard measurements made by trained healthcare providers.

In the middle of the COVID-19 pandemic, one of the most important missions in medical facilities is to protect healthcare providers from the risk of infection. The biggest difference between this highly infectious disease and other common infectious diseases, such as flu, is that COVID-19 can be fatal even in young people who do not have chronic comorbidities.^5,15^ This critical characteristic of this disease provides significant mental stress to healthcare providers. In fact, a number of healthcare providers have developed depression and were unable to continue caring for COVID-19 patients.^16,17^ As mentioned before, the careful monitoring of vital signs is crucial to avoid overlooking the deterioration due to COVID-19 even for those with mild symptoms.^18,19^ In this study, we showed that the present telemedicine-based system can provide accurate vital sign information without the risk of infection. Although the patients were in the acute phase of the disease and relatively elderly, they were able to measure their vital signs accurately and upload the data immediately using this system. One of the weaknesses of telemedicine is that elderly patients are not usually familiar with digital technologies and thus it may be difficult for hospitalised patients, who are often elderly, to use such devices. However, with our devices, which require only holding over the gateway without any procedures on the screen, all patients successfully measured and transferred the data. These results suggest that if the platform is intuitive enough, even such elderly patients can use digital technologies. Since there has been a paucity of such validation data and most similar attempts in medical facilities are started without evidence, the results of the present study are important for moving such attempts further.

#### Limitations

This study has several limitations. First, this was a pilot study conducted in a university hospital. Since patients’ digital literacy may vary depending on the area and their cultural backgrounds, the generalisability of the study should be tested in a different situation. The hospital provided a Wi-Fi network. As a stable Internet connection is crucial for providing a telemedicine system, the system may not be feasible in a medical facility where the Internet connection is unstable. The use of a portable Wi-Fi hotspot will be helpful in such places. In addition, this study does not guarantee that the use of this telemedicine system decreases the risk of infection. Further studies are necessary to elucidate the efficacy of this system in reducing infection risk and burden on healthcare providers.

## Conclusions

The use of a telemedicine-based system in the acute setting in a hospital was feasible, with all participants easily understanding the usage. Over 95% of the measurements were immediately uploaded and available on the cloud system, and the measured values demonstrated excellent agreement with the standard vital signs measured by trained healthcare providers. This system can be used as an alternative to traditional vital sign measurements by healthcare providers and the inherent risk of infection.
